# Common Variants in *OPG* Confer Risk to Bone Mineral Density Variation and Osteoporosis Fractures

**DOI:** 10.1038/s41598-017-01579-6

**Published:** 2017-05-11

**Authors:** Xiaoyong Sheng, Guangyong Cai, Xingjun Gong, Zouying Yao, Ye Zhu

**Affiliations:** 1 Taizhou Hospital of Zhejiang Province, Linhai, 317000 China; 2 Lishui Municipal Central Hospital & the Fifth Affiliated Hospital, Wenzhou Medical University, Lishui, 323000 China

## Abstract

Although many common variants have been identified for bone mineral density (BMD) and osteoporosis fractures, all the identified risk variants could only explain a small portion of heritability of BMD and osteoporosis fractures. *OPG* belongs to the tumor necrosis factor receptor superfamily, which plays a crucial role in bone remodeling and is thus a promising candidate gene of osteoporosis. Several studies have explored the association of OPG variants with BMD or osteoporosis fractures, however, the results remain inconsistent among different populations. In the study, we first assessed the relationship between OPG variants and BMD or osteoporosis fractures in our sample size (227 subjects with postmenopausal osteoporosis and 189 controls), and then performed a systematic meta-analysis. Among the nine SNPs genotyped, rs6469804 and rs2073618 showed significant associations with both BMD and osteoporotic fractures, while rs3102735 was only associated with BMD in our samples (*P* < 0.05). For meta-analyses, data for a total of 12 SNPs were pooled (4725 patients and 37804 controls), and five SNPs, including rs6993813, rs6469804, rs3134070, rs2073618 and rs3102734, showed association with osteoporosis fractures (*P* < 0.05). On light of the above analysis, we believe that OPG is one promising susceptibility gene of BMD or osteoporotic fractures.

## Introduction

Osteoporosis is a common disease which is characterized by low bone mineral density (BMD), microarchitectural deterioration of bone tissue and bone fragility^1^. An estimate of approximately 1.5 million new fracture cases was reported each year worldwide, representing huge economic burden for patient family and countries^2^. Osteoporosis is defined by clinical measurement of BMD and is the best predictor of osteoporosis fractures^3^.

Adoption, twin and family studies have revealed that the heritability of BMD is up to 50–85%^4^. Up till now, tens of hundreds of risk genes have been identified for osteoporosis and even osteoporosis fractures, through linkage analysis, candidate gene association studies and genome-wide association studies (GWAS). Most of these genes are known to influence bone physiology, specifically the reabsorption of bone by osteoclasts and the formation of bone by osteoblasts. Those genes include but not limited to *ESR1*, *COL1A1*, *LRP5*, *RANKL*, *OPG*, *SLC25A13*, etc^5–8^. Nevertheless, all the identified risk variants could only explain a small portion of heritability of osteoporosis, and many more variants remain to be identified.

*OPG* belongs to the tumor necrosis factor receptor superfamily, which plays a crucial role in bone remodeling and is thus a promising candidate gene of osteoporosis. Secreted by osteoblasts, OPG could block the RANK-RANKL signaling by competitively binding to RANKL, which could inhibit osteoclast recruitment and activation, and then induces osteoclast apoptosis^9^. Several groups have tested the association of *OPG* variation with osteoporosis susceptibility; however the results remain largely inconsistent. Studies conducted in Brazilian, American, Chinese, Slovakian samples have resulted in positive results^10–13^; while other groups using Icelandic, Australian, Brazilian and Chinese cohorts have failed to replicate this association^8, 14, 15^. Given the inconsistent association result, whether OPG variants are associated with osteoporosis remains illusive.

The heterogeneous genetic results might attribute to limited sample size used in each individual study, which may inflate the false negative and positive results simultaneously. Another possible reason would be the population heterogeneity. Many risk variants in European populations may exert protective effect for osteoporosis in Asian cohorts^12, 14^. An alternative but feasible approach is meta-analysis by combining data from a range of replication studies. In this study, we first investigated the association between *OPG* variants and BMD as well as osteoporosis fractures in an individual Chinese cohort and then performed a comprehensive meta-analysis on *OPG* variants conferring to osteoporosis fractures risk.

## Materials and Methods

### Subjects

The study consisted of 416 postmenopausal women, of which 227 were diagnosed with postmenopausal osteoporosis and 189 were healthy controls. The patients were recruited from outpatients that were admitted to the department of orthopedics from the Central Hospital of Lishui City and the People’s Hospital of Lishui City between June 2013 and September 2015. Meanwhile, healthy controls were recruited from local communities. All the subjects were of genetically unrelated Han Chinese and resided in South of China. The detailed demographic and clinical information were listed in Table 1. In general, since other chronic diseases than osteoporosis might also lead to osteoporotic fractures, subjects with chronic disorders or taking medicine were excluded from this present study^16, 17^. Written informed consent was obtained from all the enrolled subjects, and this study was approved by the Ethics Committee of the Central Hospital of Lishui City, Wenzhou Medical University. All clinical investigation was conducted according to the Declaration of Helsinki.

**Table 1:** Demographical and clinical characteristics of 416 postmenopausal women in this study.

Characteristics	Total (±SD)	Healthy group (±SD)	Osteoporosis group (±SD)	*P*-value^a^
N	416	227	189	
*N* of factures (%)	125 (30.0)	44 (19.4)	81 (42.9)	<0.001
Age (years)	65.62 (8.63)	66.11 (8.85)	65.02 (8.35)	0.201
Height (cm)	159.0 (4.88)	159.6 (4.81)	158.4 (4.90)	0.016
Weight (kg)	58.92 (9.01)	60.04 (9.16)	57.59 (8.67)	0.019
BMI (kg/m^2^)^b^	23.28 (3.24)	23.57 (3.34)	22.93 (3.08)	0.102
Age at menarche (years)	15.13 (1.79)	15.21 (1.71)	15.03 (1.89)	0.325
Age at menopause (years)	49.27 (3.85)	48.93 (3.98)	49.70 (3.66)	0.068
BMD^c^				
Lumbar spine L1–L4 BMD (g/cm^2^)	0.966 (0.154)	1.054 (0.139)	0.859 (0.092)	<0.001
Total hip BMD (g/cm^2^)	0.837 (0.136)	0.918 (0.119)	0.741 (0.083)	<0.001

^a^Healthy vs osteoporosis group; ^b^BMI, body mass index; ^c^BMD, bone mineral density.

### Measurement of bone mineral density

BMD (grams per square centimeter, g/cm^2^) at the lumbar spine (L1–L4) and total hip were measured by the dual-energy X-ray absorptiometry (DXA, GE Lunar Prodigy) for all subjects.

### SNP selection and genotyping

SNPs across the OPG gene locus were selected based on the following three criteria. First, we chose those SNPs that have been reported to be assocaited with BMD or osteoporosis fractures in other populations. Second, part of tagging SNPs were retained. In order to choose tagging SNPs, the whole SNPs within OPG genomic region were downloaded from the Hapmap project (Chinese Han Populations) and the Haploview program (version 4.1) was applied to determine the tagging SNPs using the r^2^ confidence interval (CI) algorithm^18^. The third was potential functional SNPs that might affect protein structure, mRNA expression, and so on. Finally, a total of 9 SNPs were selected for genotyping. The SNP Information was shown in Supplementary Table 1.

Genomic DNA was extracted from a total of 3 ml peripheral blood leukocytes using the standard phenol-chloroform method. Before genotyping SNPs, DNA was diluted to a final concentration of 30 ng/μl, and then all SNPs were genotyped by the TaqMan protocol as described in previous studies^19^. Genotyping assays were conducted on a CFX96 Real-Time PCR Detection System (Bio-Rad, Hercules, CA). To ensure accuracy, internal controls with known genotypes and negative controls with water were used. A total of 10% samples were randomly chosen for duplication to assess the genotyping error rate. The call rate for these SNPs was 97.4% on average and the genotype concordance was 100%.

### Study searching and data collection for meta-analyses

Assocaition between OPG variants and fractures have been extensively studied before, and meta-analyses were performed acccording to previously reported procedures^20–22^. In brief, we searched eligible studies for this meta-analysis from PubMed (http://www.ncbi.nlm.nih.gov), SCOPUS (http://www.scopus.com), EMBASE (http://www.elsevier.com/online-tools/embase) and ISIWeb of Knowledge (http://apps.webofknowledge.com/) with the following searching items “(OPG or TNFRSF11B or TR1 or OCIF or PDB5) and fractures” in text. The last search was conducted in August 12^nd^, 2016. Studies published in English were considered. We also screened the references of eligible studies for any other eligible studies.

Any studies with available genotyping data of OPG SNPs in patients with osteoporosis fractures (any location) as well as healthy controls were included. For each eligible study, two independent investigators (Sheng and Cai) extracted the following data: 1) first author and publication year; 2) fracture locations; 3) sample size and age distribution if available; 4) genotyping method; 5) genotypic and allelic distributions in cases and controls if available; and 6) P-value, odds ratio (OR) and 95% confidence interval (CI). If necessary data was unavailable, we contacted the corresponding author. The PRISMA checklist and the PRISMA flow diagram could be found in the Supplementary Materials.

### Statistical analyses

All statistical analyses were performed with the SPSS 17.0, GraphPad 5.0 and Stata 12.0 softwares. Quantitative data was represented as mean ± SD. The chi-square (χ^2^) goodness-of-fit test was applied to calculate the Hardy–Weinberg equilibrium (HWE) for each included SNP. Multiple regression and logistic regression analyses were carried out to evaluate the relationships between variables and bone mineral density as well as fractures risk, respectively.

For meta-analyses, publication bias analysis, meta-analysis, and sensitivity analysis were investigated using the Stata 12.0 software. Either Egger regression test with a funnel plot or the Begg-Mazumdar test was applied to test potential publication bias. Cochran’s χ^2^-based Q-statistic was calculated to assess the between-study heterogeneity. The pooled OR was determined by the Z-test using the random- or the fixed-effect model based on heterogeneity detection. For all test, P-value less than 0.05 was considered significant. Bonferroni correction was applied by multiplying the *P*-values by the number of tests whenever multiple tests were presented to surmount the inflation of *P*-value.

## Results

### OPG variants and BMD in our samples

A total of 9 SNPs were chosen for genotyping in our cohort, including 4 SNPs reported previously (rs6993813, rs6469804, rs3102735 and rs4355801), 3 tagging SNPs (rs7463176, rs1032128 and rs10955911) and 2 functional SNPs (rs2073617 and rs2073618). The detailed information, *i*.*e*., chromosome position, global and Chinese minor allele frequency (MAF), was listed in Supplementary Table 1. The observed allelic frequency distributions in controls for all SNPs analyzed were in Hardy–Weinberg equilibrium (*P* > 0.05). Meanwhile, based on the following assumptions: *P* = 0.05, OR = 2.00 corresponding to a “moderate to high” effect, and a given MAF according to the 1000 Genomes project (www.1000genomes.org/), the present sample size revealed more than 80% statistical power for all SNPs (Supplementary Table 1).

Table 2 showed the associations between the tested SNPs and BMD in particular locations. Three SNPs, namely rs6469804, rs3102735 and rs2073618, exhibited significant association with BMD in either Lumbar spine L1–L4 or total hip after adjusting for age, BMI, and age at menopause. Specifically, rs6469804 correlated with BMD with a *P*-value of 0.048 for Lumbar spine L1–L4 (β = −0.07, 95% CI = −0.23 to 0.00), and a *P*-value of 0.012 for total hip (β = −0.09, 95% CI = −0.25 to −0.04). While rs3102735 was only associated with total hip BMD (*P* = 0.006, β = −0.10, 95% CI = −0.28 to −0.07), and rs2073618 only with Lumbar spine L1–L4 (*P* = 0.015, β = −0.08, 95% CI = −0.30 to −0.04). After stringent Bonferroni correction, none of the significance remained (*P*-value for significance was set at 0.006).

**Table 2:** Association of 9 SNPs with BMD at Lumbar spine L1–L4 and total hip.

SNP	Minor allele	Risk Allele	RAF^a^	Lumbar spine L1–L4			Total hip		
				Standardized coefficients β	β 95% CI	*P*-value^b^	Standardized coefficients β	β 95% CI	*P*-value
rs6993813	T	C	0.610	−0.04	−0.15, 0.04	0.203	−0.04	−0.20, 0.03	0.187
rs6469804	G	A	0.791	−0.07	−0.23, 0.00	0.048	−0.09	−0.25, −0.04	0.012
rs3102735	C	T	0.878	−0.03	−0.09, 0.02	0.127	−0.10	−0.28, −0.07	0.006
rs2073617	G	G	0.415	−0.04	−0.19, 0.05	0.244	−0.01	−0.07, 0.12	0.831
rs2073618	G	C	0.642	−0.08	−0.30, −0.04	0.015	−0.03	−0.16, 0.04	0.209
rs7463176	A	G	0.674	−0.01	−0.21, 0.14	0.906	0.00	−0.10, 0.11	0.739
rs1032128	A	A	0.407	−0.02	−0.16, 0.09	0.583	−0.05	−0.20, 0.01	0.083
rs10955911	T	T	0.079	0.00	−0.17, 0.18	0.942	−0.02	−0.18, 0.07	0.458
rs4355801	G	A	0.695	−0.02	−0.16, 0.08	0.528	0.00	−0.08, 0.10	0.695

^a^RAF, risk allele frequency. ^b^ *P*-value of significance was set at 0.006 by using the Bonferroni Correction.

### OPG variants and osteoporotic fractures in our samples

Table 3 showed the associations of each SNP with osteoporotic fractures. Corresponding to the association results of BMD, after adjusting for age, BMI, and age at menopause, rs6469804 (*P* = 0.026, OR = 0.656. 95% CI = 0.451–0.952) and rs2073618 (*P* = 0.039, OR = 0.721, 95% CI = 0.528–0.984) also showed significant associations with osteoporotic fractures, and the minor allele (rs6469804-G and rs2073618-G) showed protective effect for osteoporotic fracture onset. However, neither rs6469804 (*P* = 0.234) nor rs2073618 (*P* = 0.351) survived for Bonferroni correction (*P*-value for significance was set at 0.006).

**Table 3:** Association of 9 SNPs with osteoporotic fracture.

SNP	Minor allele	MAF		OR	95% CI	*P*-value^a^
		Cases	Controls			
rs6993813	T	0.384	0.399	0.940	0.694–1.275	0.692
rs6469804	G	0.180	0.251	0.656	0.451–0.952	0.026
rs3102735	C	0.112	0.137	0.791	0.500–1.252	0.317
rs2073617	G	0.417	0.412	1.015	0.751–1.371	0.922
rs2073618	G	0.328	0.401	0.721	0.528–0.984	0.039
rs7463176	A	0.324	0.330	0.974	0.710–1.336	0.868
rs1032128	A	0.416	0.395	1.090	0.806–1.474	0.575
rs10955911	T	0.088	0.067	1.343	0.779–2.317	0.287
rs4355801	G	0.300	0.313	0.942	0.682–1.300	0.716

^a^ *P*-value of significance was set at 0.006 by using the Bonferroni Correction.

### Meta-analyses of the associations between OPG variants and osteoporotic fracture

**Figure 1:**
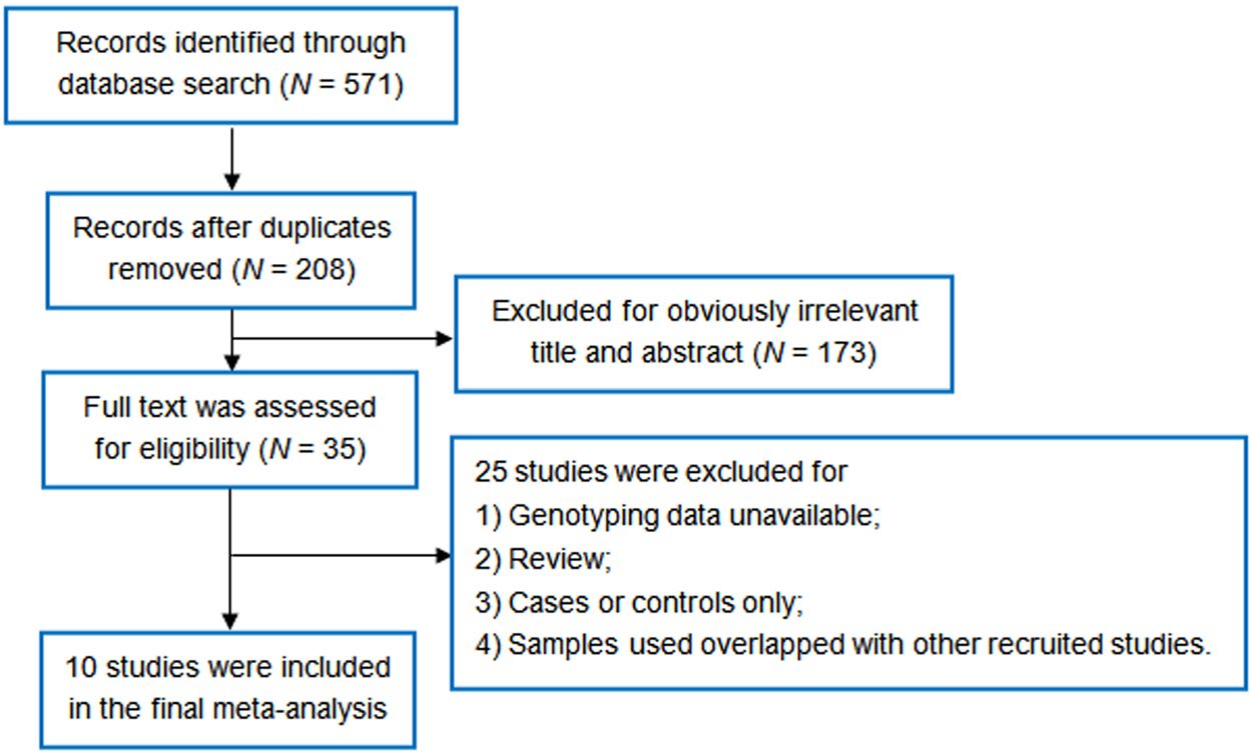
Literature search flow chart.

According to our literature search approaches, a total of 208 references were indexed, among which 198 studies were excluded for one or more of the following reasons: 1) genotyping data available, 2) samples overlapping with other studies, 3) review, and 4) controls or cases only (Fig. 1). Finally, a total of 10 studies were enrolled in this meta-analysis^8, 10–15, 23–25^, and a total of 12 SNPs with genotyping data in at least one study were meta-analyzed. Supplementary Table 2 listed the detailed information of recruited studies for this meta-analysis, *i*.*e*., populations, sample size, number of SNPs, age and sex distribution, *etc*. Supplementary Table 3 listed the information of SNPs for meta-analysis, including chromosome position, alleles and MAF.

**Figure 2:**
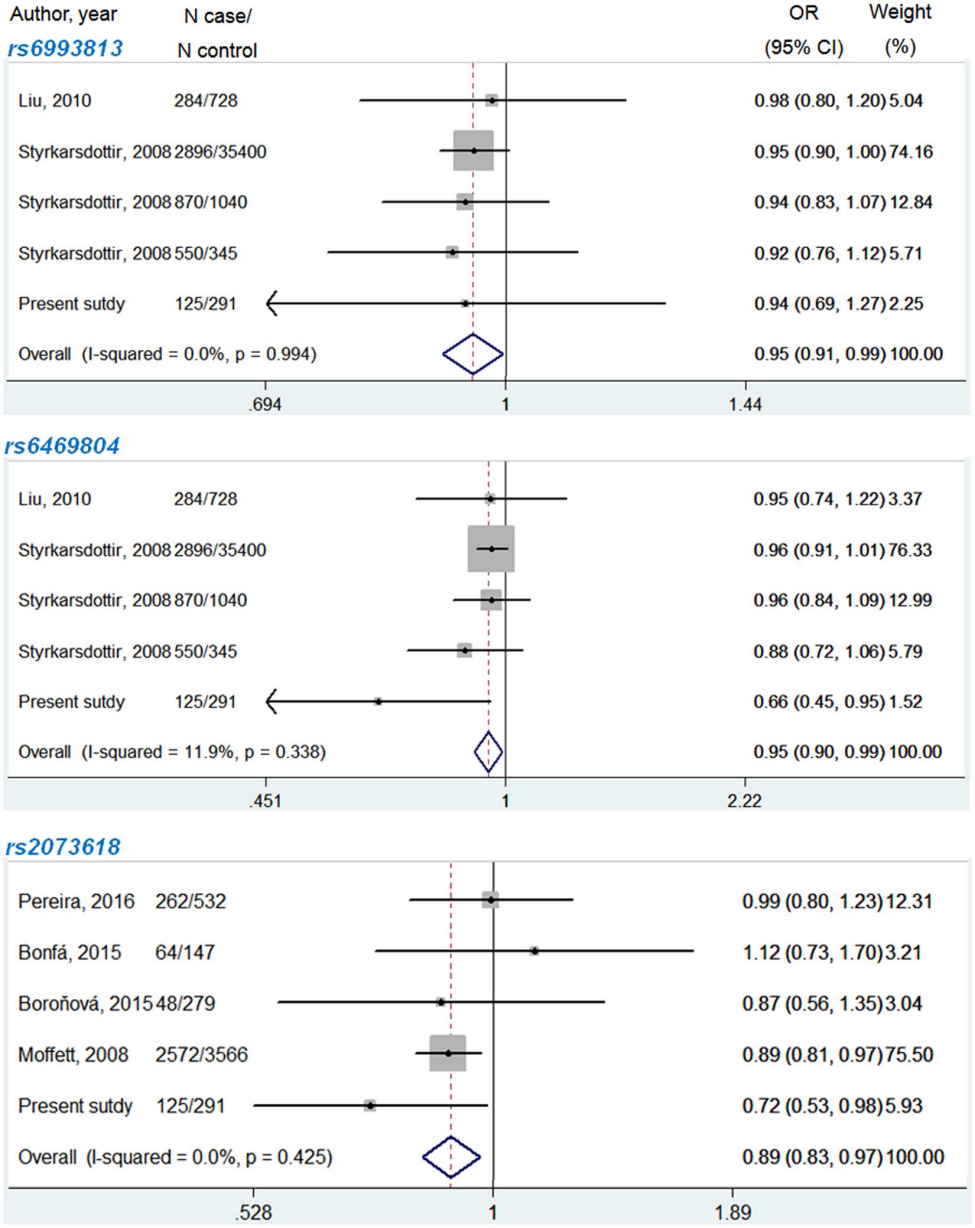
Forest plot of meta-analysis for rs6993813, rs6469804 and rs2073618 of the OPG gene using fixed-effect model.

Among the 12 SNPs, 7 SNPs had genotyping data in at least two studies, including rs6993813, rs6469804, rs3102735, rs2073617, rs2073618, rs3134069 and rs4355801 (Table 4). Rs6993813 and rs6469804 were genotyped in a total of 5 populations (4725 patients and 37804 controls) from 3 studies, including the present study^14, 15^. Since no obvious between-study heterogeneity was observed for rs6993813 (I^2^ = 0.00%, *P* = 0.994) and rs6469804 (I^2^ = 11.90%, *P* = 0.338), the fixed model was applied to pool data. Meta-analyses showed that both rs6993813 (*P* = 0.029, OR = 0.95, 95% CI = 0.91–0.99) and rs6469804 (*P* = 0.019, OR = 0.95, 95% CI = 0.90–0.99) were significantly associated with osteoporotic fractures (Fig. 2). For rs2073618, 5 studies encompassing 3071 cases and 4815 controls were pooled by the fixed model meta-analysis (I^2^ = 0.00%, *P* = 0.425), and it was found that rs2073618 was significantly associated with osteoporotic fractures (*P* = 0.004, OR = 0.89, 95% CI = 0.83–0.97) (Fig. 2). Moreover, rs3102734 (*P* = 0.004, OR = 0.89, 95% CI = 0.83–0.97) and rs2073618 (*P* = 6.3E-05, OR = 1.38, 95% CI = 1.22–1.58) were associated with osteoporotic fractures with the lowest *P*-value. Among all the nominally associated SNPs, only rs2073618 and rs3102734 survived for Bonferroni correction (*P*-value for significance was set at 0.004).

**Table 4:** Statistics of meta-analyses for all included *OPG* SNPs.

SNP	Major/minor allele	No. of comparisons	Sample size		Heterogeneity		Publication bias (P-value)^a^	Model	Meta-analyses		
			Patients	Controls	*I* ^b^	*P*-value			Pooled OR	95% CI	*P*-value^b^
rs6993813	C/T	**5**	**4725**	**37804**	**0**.**00%**	**0**.**994**	**0**.**653**	**Fixed**	**0**.**95**	**0**.**91–0**.**99**	**0**.**029**
rs6469804	A/G	**5**	**4725**	**37804**	**11**.**90%**	**0**.**338**	**0**.**161**	**Fixed**	**0**.**95**	**0**.**90–0**.**99**	**0**.**019**
rs11995824	G/C	1	735	277	n.a.	n.a.	n.a.	n.a.	1.01	0.82–1.24	0.920
rs3102735	C/T	4	769	979	63.30%	0.043	0.172	Random	0.98	0.68–1.42	0.921
rs3134070	G/A	**1**	**532**	**262**	**n**.**a**.	**n**.**a**.	**n**.**a**.	**n**.**a**.	**1**.**52**	**1**.**09–2**.**12**	**0**.**012**
rs34353469	T/G	1	247	152	n.a.	n.a.	n.a.	n.a.	0.89	0.65–1.23	0.484
rs2073617	T/C	3	1532	3091	0.00%	0.408	0.649	Fixed	1.06	0.92–1.22	0.394
rs2073618	C/G	**5**	**3071**	**4815**	**0**.**00%**	**0**.**425**	**0**.**847**	**Fixed**	**0**.**89**	**0**.**83–0**.**97**	**0**.**004**
rs3102734	G/A	**1**	**1046**	**2303**	**n**.**a**.	**n**.**a**.	**n**.**a**.	**n**.**a**.	**1**.**38**	**1**.**22–1**.**58**	**6**.**3E-05**
rs3102733	T/C	1	247	152	n.a.	n.a.	n.a.	n.a.	0.8	0.63–1.18	0.362
rs3134069	T/G	3	644	688	73.80%	0.022	0.648	Fixed	1.74	0.97–3.14	0.063
rs4355801	A/G	2	409	1019	0.00%	0.757	0.824	Fixed	0.98	0.83–1.16	0.849

^a^Results of Egger test; ^b^ *P*-value of significance was set at 0.004 by using the Bonferroni Correction. Supplementary materials included Tables S1–S3, PRISMA checklist and PRISMA flow chart.

## Discussion

Like other genetically complex diseases, osteoporosis is the results of combinational effect of multiple genetic and environmental factors. Several studies have proved that genetic risk factors play a crucial role in the pathogenesis of osteoporosis with an estimated heritability of 55–85%^26, 27^. By applying large-scale GWAS, many susceptibility loci conferring risk to BMD have been identified. For example, one recent published GWAS using European and east Asian cohorts identified 56 loci associated with BMD which surpassed genome-wide significance (*P* < 5E-08), and 14 SNPs among them showed significant association with fracture risk^7^. However, candidate association studies of BMD or osteoporotic fracture focusing on bone remodeling related genes have produced conflicting results in different populations^8, 15^. Discordant results may be due to different populations or limited sample size used in previous studies. Success of genetic analysis of BMD and osteoporosis fracture will require either large sample sizes by meta-analysis.

In the present study, we first tested a total of 9 SNPs across the OPG locus which would influence the BMD and osteoporotic fracture in Chinese women, and found that 3 SNPs (rs6469804, rs3102735 and rs2073618) were associated with either spine or hip BMD after adjusting for other confounding facators, *i*.*e*., age, BMI, and age at menopause, further suggesting that common variants in OPG variation might contribute to BMD variation and even osteoporotic fracture. In order to validate our result, we then performed a comprehensive meta-analysis of the associations between OPG variation and osteoporotic fracture with the largest sample size to date (4725 patients and 37804 controls). In line with our expectations, a total of 5 SNPs showed significant association with osteoporotic fracture with the lowest *P*-value for rs3102734 (*P* = 6.3E-05).

With a key role in bone remodeling, OPG could bind to RANKL, a protein expressed on the osteoblast surface, thus blocking the interaction between RANKL and RANK on the osteoclast membrane. The *OPG-RANKL* interaction might exert antiresorptive effect in bone remodeling^9^. *In vivo* experiments also demonstrated that *OPG* knockout mice could develop severe osteoporosis^28^. OPG has been shown to be associated with osteoporosis fracture in various ethnicities. In a recent multi-ethnicity GWAS with more than 130000 cases and controls, authors found that SNPs in genes of the RANK-RANKL-OPG pathway despite showed the strongest BMD-associated signal^7^.

We could notice that not all variants that affect BMD are associated with osteoporosis fracture. As a matter of fact, only a small portion of BMD-related loci also show significant association with osteoporosis fracture. This might be not surprising when we take into consideration the following several reasons. First, although lower BMD is a prerequisite for fracture, only lower BMD combined with other risk factors could lead to osteoporotic fractures. Second, fracture is the extreme condition of osteoporosis.

Inconsistent with previous studies that enrolled men and women at the same time, we enrolled premenopausal patients only since it had been reported that estrogen deficiency might lead to bone loss and then confound the association studies^29^. This is one advantage of our study. There are, however, several limitations to the interpretation of our study. Firstly, although the combined sample size was large, most of the samples were from European districts, while Chinese samples account for only a small portion, which may contribute to type II errors of our results. Secondly, we focused only on the common variants in the OPG locus, and whether rare mutations in OPG also confer risk to BMD and even osteoporotic fractures remains largely unknown. Future study would focus more on rare variants of candidate genes through genome and candidate region sequencing. Thirdly, except for rs3134070 and rs3102734, the effect size for other risk variants were too small, and they might not represent causal variants, but only association signals which were in LD with other true causal variants. Fourthly, meta-analyses of the associations between OPG variants and BMD were not performed since the data necessary for meta-analyses was unavailable in most of studies. In this case, the meta-analytic results might be inaccurate since sample variance couldn’t be deduced from the original manuscript.

## Conclusion

In light of the above analysis, we believe that OPG is one promising susceptibility gene of BMD or osteoporotic fractures. However, whether rare variants also confer risk to BMD or osteoporotic fractures remain to be identified.

## Electronic supplementary material

Supplementary materials
