# Supplementary material for: Common Variants in OPG Confer Risk to Bone Mineral Density Variation and Osteoporosis Fractures
Source: Sci Rep. 2017 May 11;7:1739. doi: 10.1038/s41598-017-01579-6 (PMC5432005; doi:10.1038/s41598-017-01579-6)
Supplement: Supplementary file 1 — Supplementary materials [file 41598_2017_1579_MOESM1_ESM.doc]

**Supplementary materials for:**

**Common Variants in *OPG* Confer Risk to Bone Mineral Density Variation and Osteoporosis Fractures**

Xiaoyong Sheng^1, *^, Guangyong Cai^2^, Xingjun Gong^2^, Zouying Yao^2^, Ye Zhu^2^

^1^Taizhou Hospital of Zhejiang Province, Linhai, 317000, China

^2^Lishui Minicipal Central Hospital & the Fifth Affiliated Hospital, Wenzhou Medical University, Lishui, 323000, China

* Correspondence to:

Dr. Ye Zhu (email: [zhuye1987@163.com](mailto:zhuye1987@163.com))

Lishui Minicipal Central Hospital & the Fifth Affiliated Hospital, Wenzhou Medical University, No. 289, Kuocang Road, Lishui, 323000, China

**Contents**

[Supplementary Table 1 ………………………………………………………..3](#__RefHeading___Toc437334868)

**PR**ISMA flow chart………………………………………………..………….………4

**Supplementary Table 2………………………………………………………...………5**

Supplementary Table 3………………………………………………………...………7

**Table S1 Information of OPG SNPs selected for genotyping**

| #SNP | rs ID | Position^1^ | Major/minor allele | Global MAF^2^ | MAF^2^  (*Chinese Han*) | HWE^3^  *P*-value | Annotation | Statistical  Power^4^ |
| --- | --- | --- | --- | --- | --- | --- | --- | --- |
| 1 | rs6993813 | 119039999 | C/T | 0.353 | 0.407 | 0.325 | Reported | 93.5% |
| 2 | rs6469804 | 119032590 | A/G | 0.252 | 0.244 | 0.258 | Reported | 90.5% |
| 3 | rs3102735 | 118952831 | T/C | 0.167 | 0.134 | 0.726 | Reported | 82.5% |
| 4 | rs2073617 | 118952044 | A/G | 0.378 | 0.444 | 0.193 | 5’ UTR | 93.8% |
| 5 | rs2073618 | 118951813 | C/G | 0.333 | 0.341 | 0.207 | Missense mutation | 93.2% |
| 6 | rs7463176 | 118945386 | G/A | 0.270 | 0.344 | 0.492 | Tagging | 91.4% |
| 7 | rs1032128 | 118939534 | G/A | 0.387 | 0.430 | 0.317 | Tagging | 93.8% |
| 8 | rs10955911 | 118925502 | C/T | 0.149 | 0.089 | 0.067 | Tagging | 80.3% |
| 9 | rs4355801 | 118911634 | A/G | 0.276 | 0.349 | 0.322 | Reported | 91.6% |

^1^Position was based on hg19.

^2^MAF, minor allele frequency in global and Chinese Han populations, respectively.

^3^HWE, Hardy–Weinberg equilibrium in healthy subjects.

^4^Statistical power was calculated based on the following assumptions: *P* = 0.05, OR = 2.00 corresponding to a “moderate to high” effect, and the global MAF of each SNP.


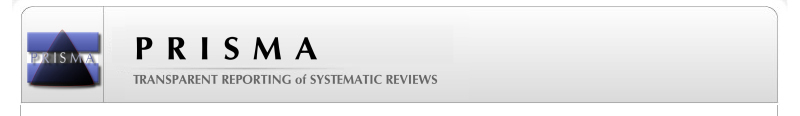
**PRISMA 2009 Flow Diagram**

**Screening**

**Included**

**Eligibility**

**Identification**

Records identified through database searching
(n = 571 )

Additional records identified through other sources
(n = 0 )

Records after duplicates removed
(n = 208 )

Records screened
(n = 208 )

Records excluded
(n = 173 )

Full-text articles assessed for eligibility
(n = 35 )

Full-text articles excluded, with reasons
(n = 25 )

Studies included in qualitative synthesis
(n = 10 )

Studies included in quantitative synthesis (meta-analysis)
(n = 10 )

*From:* Moher D, Liberati A, Tetzlaff J, Altman DG, The PRISMA Group (2009). *P*referred *R*eporting *I*tems for *S*ystematic Reviews and *M*eta-*A*nalyses: The PRISMA Statement. PLoS Med 6(7): e1000097. doi:10.1371/journal.pmed1000097

**For more information, visit www.prisma-statement.org.**

**Table S2 Characteristics of included studies in the present meta-analysis**

| Author, year | Ethnicity | Fracture locations | Case | Case age | Control | Control age | Title |
| --- | --- | --- | --- | --- | --- | --- | --- |
| Pereira, 2016 | Brazil | Vertebral | 262 | n.a. | 532 | n.a. | Associations between OPG and RANKL polymorphisms, vertebral fractures, and abdominal aortic calcification in community-dwelling older subjects: the Sao Paulo Ageing & Health Study (SPAH) |
| Bonfá, 2015 | Brazil | Vertebral | 64 | 35.6 (7.3) | 147 | 32.3 (7.0) | RANKL and OPG gene polymorphisms: associations with vertebral fractures and bone mineral density in premenopausal systemic lupus erythematosus |
| Boroňová, 2015 | Slovakia | n.a. | 48 | 66.92 (9.63) | 279 | 64.68 (9.18) | TNFRSF11B gene polymorphisms, bone mineral density, and fractures in Slovak postmenopausal women |
| Deng, 2013 | China | n.a. | 735 | n.a. | 277 | n.a. | The influence of the genetic and non-genetic factors on bone mineral density and osteoporotic fractures in Chinese women |
| Song, 2013 | China | n.a. | 247 | 49-80 | 152 | 48-81 | Association between single nucleotide polymorphisms of the osteoprotegerin gene and postmenopausal osteoporosis in Chinese women |
| Wang, 2012 | China | Nontraumatic fractures | 1046 | 69.74 (8.85) | 2303 | 64.65 (7.19) | Susceptibility Genes for Osteoporotic Fracture in Postmenopausal Chinese Women |
| Liu, 2010 | China | n.a.^1^ | 284 | 60.05 (10.47) | 728 | 54.81 (11.20) | Analysis of Recently Identified Osteoporosis Susceptibility Genes in Han Chinese Women |
| Moffett, 2008 | America | Hip, Femoral, Intertrochanteric, Wrist | 2572 | n.a. | 3566 | n.a. | Osteoprotegerin Lys3Asn Polymorphism and the Risk of Fracture in Older Women |
| Styrkarsdottir, 2008 | Iceland | Forearm, Hip, Vertebral | 2896 | n.a. | 35400 | n.a. | Multiple Genetic Loci for Bone Mineral Density and Fractures |
|  | Denmark |  | 870 | n.a. | 1040 | n.a. |  |
|  | Australia |  | 550 | n.a. | 345 | n.a. |  |
| Brandstrom, 2004 | Europe | Hip, Wrist, Vertebral | 361 | n.a. | 497 | n.a. | Single Nucleotide Polymorphisms in the Human Gene for Osteoprotegerin are Not Related to Bone Mineral Density or Fracture in Elderly Women |

^1^n.a., data not available.

**Table S3 Information of OPG SNPs included in the present meta-analysis**

| #SNP | rs ID | Position^1^ | Major/minor allele | Global MAF^2^ | MAF^2^  (*Chinese Han*) | Annotation |
| --- | --- | --- | --- | --- | --- | --- |
| 1 | rs6993813 | 119039999 | C/T | 0.353 | 0.407 | Upstream |
| 2 | rs6469804 | 119032590 | A/G | 0.252 | 0.244 | Upstream |
| 3 | rs11995824 | 119000461 | G/C | 0.322 | 0.400 | Upstream |
| 4 | rs3102735 | 118952831 | T/C | 0.167 | 0.134 | Upstream |
| 5 | rs3134070 | 118952785 | C/T | 0.107 | 0.105 | Upstream |
| 6 | rs3134069 | 118952749 | A/C | 0.107 | 0.105 | Upstream |
| 7 | rs2073617 | 118952044 | A/G | 0.378 | 0.444 | 5’ UTR |
| 8 | rs2073618 | 118951813 | C/G | 0.333 | 0.341 | Exon 1 |
| 9 | rs3102734 | 118951777 | G/A | 0.105 | 0.105 | Intron 1 |
| 10 | rs3102733 | 118949881 | T/C | 0.105 | 0.100 | Intron 1 |
| 11 | rs34353469 | 118924469 | T/G | n.a. | n.a. | Exon 5 |
| 12 | rs4355801 | 118911634 | A/G | 0.276 | 0.349 | Reported |

^1^Position was based on hg19.

^2^MAF, minor allele frequency in global and Chinese Han populations, respectively.
